# Bio-spectroscopic investigation linking changes of retinal structure with short-term administration of Amiodarone and revealing the ameliorative effect of vitamin E supplementation

**DOI:** 10.1038/s41598-024-70573-6

**Published:** 2024-09-05

**Authors:** Sherif S. Mahmoud, Sahar A. Morsy, Eman M. Aly, Islam A. Mohalhal

**Affiliations:** 1 Biophysics and Laser Science Unit, Research Institute of Ophthalmology, Giza, Egypt; 2 Physics Department, Faculty of Science, Al-Azhar University (Girls Branch), Cairo, Egypt; 3 Retina Department, Research Institute of Ophthalmology, Giza, Egypt

**Keywords:** Retina, Fundus, FTIR, Amiodarone, Vitamin E, Biophysics, Medical research

## Abstract

Long term use of Amiodarone (AMIO) is associated with the development of ocular adverse effects. This study investigates the short term effects, and the ameliorative consequence of vitamin E on retinal changes that were associated with administration of AMIO. This is accomplished by investigating both retinal structural and conformational characteristics using Fourier transform infrared spectroscopy (FTIR) and Fundus examination. Three groups of healthy rabbits of both sexes were used; the first group served as control. The second group was orally treated with AMIO (160 mg /kg body weight) in a daily basis for two weeks. The last group orally received AMIO as the second group for two weeks then, oral administration of vitamin E (100 mg/kg body weight) for another two weeks as well. FTIR results revealed significant structural and conformational changes in retinal tissue constituents that include lipids and proteins due to AMIO administration. AMIO treatment was associated with fluctuated changes (increased/decreased) in the band position and bandwidth of NH, OH, and CH bonds. This was concomitant with changes in the percentage of retinal protein constituents in particularly α-helix and Turns. AMIO facilitates the formation of intra-molecular hydrogen bonding and turned retinal lipids to be more disordered structure. In conclusion, the obtained FTIR data together with principal component analysis provide evidence that administration of vitamin E following the treatment with AMIO can ameliorate these retinal changes and, these biophysical changes are too early to be detected by Fundus examination.

## Introduction

Since its introduction in 1960 as a powerful coronary vasodilator for treating anginal symptoms, Amiodarone (AMIO) is still the most effective drug for the treatment of arrhythmia. In one hand, it was labeled by the US-FDA for the treatment of life-threatening ventricular arrhythmias in 1985^1–3^. On the other hand, it is used off-label to treat atrial fibrillation as well as for the prevention of ventricular tachyarrhythmia in high-risk patients^2^. The mechanism of action of AMIO comes from the alteration of the function of many membrane protein ion channels, ion exchangers, and adrenergic receptors, which lead to prolongation of the action potential duration of atrial and ventricular muscles without altering the resting membrane potential; therefore, this contributes to complex therapeutic and toxicity profiles^3^. Because of the long elimination half-life of AMIO and its effect on multiple ion channels and receptors, the toxicity profiles include pulmonary^4^, renal^5^ genital^6^, liver^7^_,_ thyroid dysfunction^8^ and peripheral neuropathy^9^.

The dominance of Amiodarone therapy-adverse effects reaches 15% at the first year and 50% for long-term^2^. Ocular side effects associated with AMIO were firstly reported in 1969^10^ and include corneal microdeposits^11^ in at least 90% of patients^12,13^, lens opacities without visual impairment^11^, Optic neuropathy and retinopathy^14^, abnormal phospholipid accumulation in the retinal pigment epithelial cells and induced apoptosis^15^. Although Amiodarone-induced retinopathy is rare and the main cause of visual loss is due to optic neuropathy during long-term AMIO therapy^16^, histopathological studies indicate that intra-cytoplasmic deposits of AMIO were detected in retinal pigment epithelial cells and ganglion cells as well; in addition to cornea, lens and optic nerve^17^. These deposits in particularly that located in the retina was reported to act as photosensitizing agent and results in retinal phototoxicity. Joshi and Gill (2017) reported the first case with Amiodarone induced retinal phototoxicity following vitrectomy surgery. A 66-year-old male was on oral Amiodarone and developed retinal phototoxicity from intraoperative light exposure.

This study reports for the first time, to the best of knowledge, the effects of short term oral administration (two weeks) of Amiodarone on vibrational characteristics of retina and the potential effect of Vitamin E (Vit. E) post-administration. After ophthalmic examination, retinal characteristics were investigated by Fourier transform infrared spectroscopy (FTIR) and FTIR-data were statistically evaluated by principal component analysis as well.

## Results

### Ophthalmic examination

**Fig. 1:**
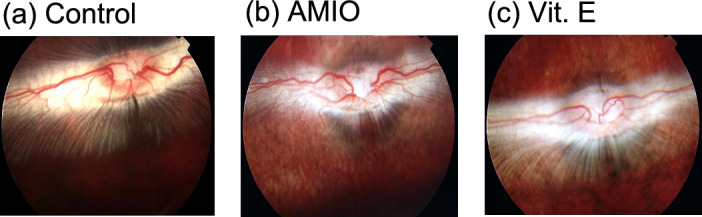
Fundus examination of retinae; (**a**) Control, (**b**) AMIO group, and (**c**) Vit. E group.

In Fig. 1.a, examination of the control rabbit retina showed optic nerve head, choroid and retinal blood vessels. The optic nerve head is orange-red in color and oval in shape. Retinal blood vessels and myelinated nerve fibers were crossing the retina in a horizontal plane from the optic disc. Anatomically, and by morphology retinal arteries were thinner than retinal veins^18^. Choroid was visible under the retina, but there was no distinguishable macular like structures. The optic disc is situated above the horizontal midline of the eye so during examination of the retina we have to look upwards. There were no clinical changes in the retinae that were treated with Amiodarone for two weeks and those post-administered with Vit. E (Fig. 1b,c).

### Infrared spectroscopy

Detailed analysis of the FTIR spectra was carried out for the following three ranges: 4000 -3000 cm^-1^ (NH-OH region), 3050—2800 cm^−1^ (CH stretching region), and 1800—900 cm^-1^ (fingerprint region) with special consideration of the bands at 1800—1595 cm^-1^ (lipid carbonyl and protein Amide I absorption bands).

### NH-OH region

**Fig. 2:**
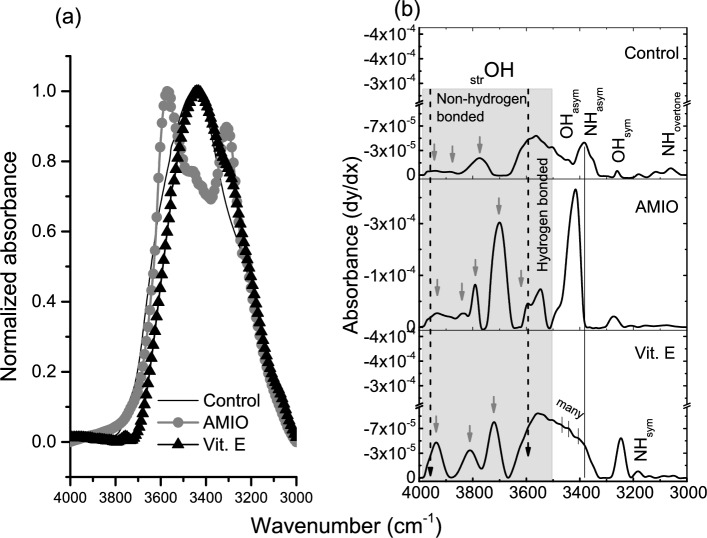
Normalized-FTIR spectra of the NH-OH region of the control retinae and the other groups that received AMIO either alone or with Vit. E (**a**), and their second derivative spectra (**b**).

The stretching NH-OH region of the control and the treated animals in the absorption range 4000–3000 cm^−1^ is shown in Fig. 2. After normalizing the existing data (panel a), the second derivative spectra are displayed in panel (b). The overlaid spectra in panel (a) clearly show that the pattern of AMIO group is different than the pattern of both control and Vit. E groups and, there are some similarities between the last two groups. The differentiated spectra in panel (b) indicate that AMIO treatments increase the absorption intensity not only that, but also the discrepancy in results is more pronounced. In AMIO group, the non-hydrogen bonded OH groups in the range 4000 – 3650 cm^−1^ show five absorption bands (arrows) with two strong absorptions at 3702 and 3588 cm^−1^ and, another absorption band at 3563 cm^-1^ that belongs to OH-hydrogen bonded group. A strong absorption due to OH_asym_ is also obvious in AMIO group.

Comparing the control pattern with that of Vit. E group; the main absorption in the non-hydrogen bonded-OH group is noticed at 3625 cm^−1^ for the control while; it located at 3756 cm^−1^ in Vit. E group. The absorption pattern of both groups is similar in the frequency range 3580–3400 cm^−1^ which comprises both hydrogen bonded OH group and asymmetric OH vibrations. On the other hand, OH_sym_ mode was characterized by higher intensity in Vit. E group relative to the control one. NH symmetric stretching mode was found to be associated with Vit. E group only.

### CH region

**Fig. 3:**
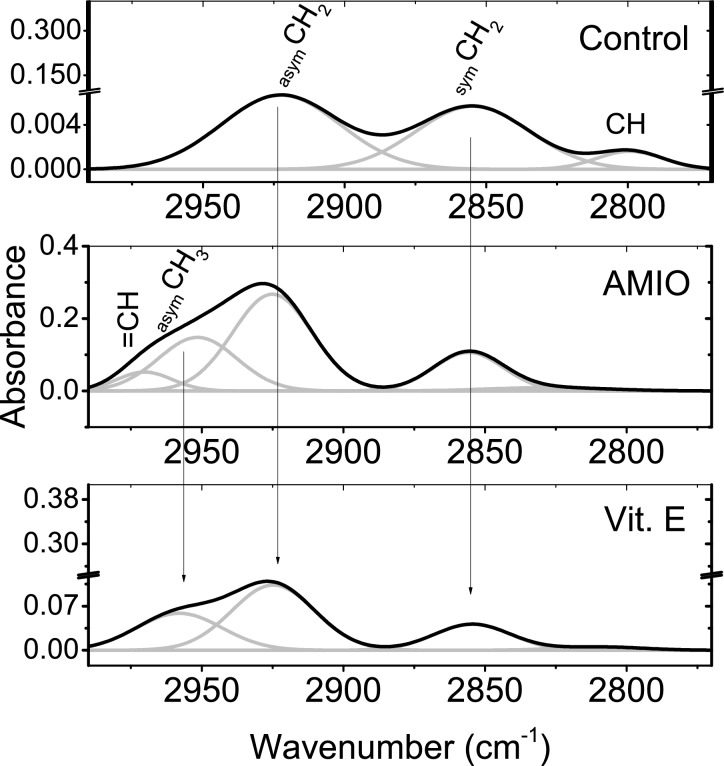
FTIR spectra related to the CH stretching region of the control retinae and the treated groups. The constituting bands are displayed in gray lines.

The band fitting of the CH stretching vibrations in the range 3000 – 2800 cm^−1^ is displayed in Fig. 3. These vibrations are associated with retinal lipids (νCH_2_) and proteins (_sym_CH_3_, if any). The control pattern revealed the presence of three underlying bands at 2920 cm^−1^ (_asym_CH_2_), 2854 cm^-1^ (_sym_CH_2_) and 2800 cm^-1^ (CH). The absorption pattern of AMIO group show more underlying bands where; unsaturated CH band (olefinic = CH, 2970 cm^−1^) and _asym_CH_3_ (2951 cm^-1^) vibrations were detected. Asymmetric CH_2_ mode of vibration also influenced by AMIO administration where; its band position is increased concomitant with reduced band width. In addition, the unidentified CH mode of vibration was characterized by increased band position associated with increased bandwidth. Although _asym_CH_3_ vibrational mode is detected in Vit. E group, the rest of vibrational bands matches their control values.

Table 1 displays these variations and their significance as well. The band area ratio of _asym_CH_2_/_sym_CH_2_ was calculated and found to be increased from 1.3 in the control to 2.8 in both AMIO and Vit. E groups.

**Table 1:** Band fitting analysis of CH stretching region of control and treated groups.

	= CH	_asym_CH_3_	_asym_CH_2_	_sym_CH_2_	CH
Control			2920 ± 2 49 ± 4	2854 ± 2 42 ± 6	2800 ± 3 28 ± 5
AMIO group	2970 ± 2 23 ± 5	2951 ± 2 33 ± 4	^†^2925 ± 1 ^†^33 ± 4	2855 ± 2 33 ± 3	^†^2827 ± 2 ^†^49 ± 3
Vit. E group		2957 ± 3 36 ± 5	2921 ± 1 41 ± 4	2854 ± 2 35 ± 4	2801 ± 3 33 ± 4

First line in each cell displays the band position (cm^-1^) and, second line displays bandwidth (cm^-1^).

^†^Statistically significant.

### Fingerprint region

**Fig. 4:**
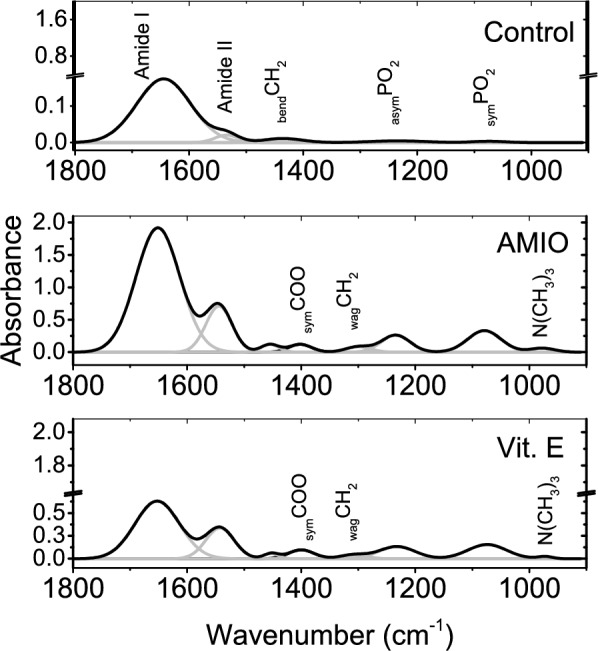
Fingerprint region of the control retinae and the treated groups. Underlying bands are displayed in grey lines.

The fingerprint region (1800—900 cm^−1^) results from the absorption of functional groups relate to all retinal constituents. In Fig. 4, and as compared to the control pattern, the absorption pattern of AMIO group is characterized by increased absorption intensity, and administration of Vit. E reduces this absorption intensity.

The band position of both Amide I and Amide II was upshifted due to AMIO treatment. The bending mode of CH_2_ group was found to be affected by AMIO treatment; upshifted band position associated with decreased band position. These two observations are quite similar to Vit. E group. Regarding the phosphate group vibrations; the changes were noticed only for _asym_PO_2_ where, the band width was reduced in AMIO as well as Vit. E groups. More vibrational bands were detected in AMIO and Vit. E groups namely; _sym_COOC, wagging CH_2_ and (CH_3_)_3_N. Comparing the last mentioned vibrational band of both treated groups; it is noticed that Vit. E administration reduces the band characteristics; position and width. Table 2 summarizes all these values and their statistical significance as well.

**Table 2:** Fingerprint region band characteristics of control and treated groups.

	Control	AMIO	Vit. E
Amide I	1644 ± 2 111 ± 10	^†^1651 ± 2 91 ± 8	^†^1652 ± 1 92 ± 11
Amide II	1534 ± 1 49 ± 6	^†^1544 ± 3 54 ± 5	^†^1542 ± 2 58 ± 6
_bend_CH_2_	1436 ± 2 80 ± 4	^†^1455 ± 3 ^†^37 ± 3	^†^1453 ± 3 ^†^31 ± 5
_sym_COOC		1401 ± 2 49 ± 3	1399 ± 1 53 ± 3
_wag_CH_2_		1303 ± 3 51 ± 4	1304 ± 3 54 ± 6
_asym_PO_2_	1234 ± 3 116 ± 8	1234 ± 2 ^†^59 ± 5	1232 ± 2 ^†^74 ± 7
_sym_PO_2_	1074 ± 3 70 ± 5	1077 ± 4 68 ± 4	1074 ± 4 78 ± 6
(CH_3_)_3_N		977 ± 1 52 ± 2	972 ± 2 31 ± 4

First line in each cell displays the band position (cm^-1^) and, second line displays bandwidth (cm^-1^).

^†^Statistically significant.

Ratiometric analysis of Amide I/II absorption intensity revealed difference in its value for all groups; in the control, this value was calculated and found to be 8.7 while; it reduced to 2.7 and 0.2 for AMIO and Vit. E groups respectively. The intensity ratio of PO_2_ sym/asym bands was also calculated and its value for the control is 0.7 while in AMIO and Vit. E groups it was 1.3 and 1.2 respectively.

### Carbonyl bands

**Fig. 5:**
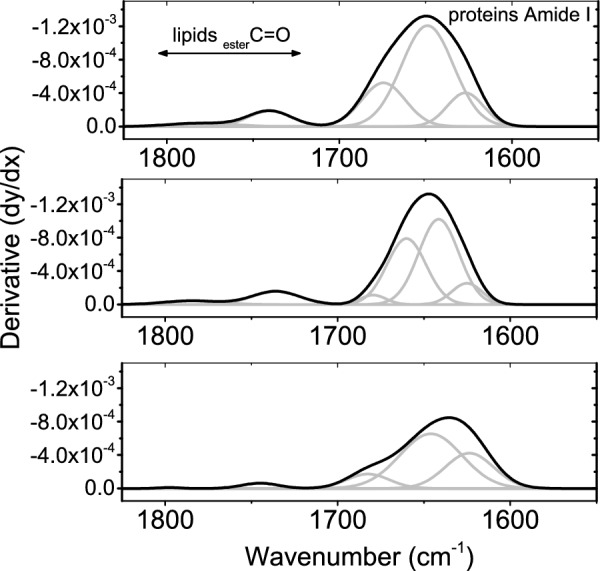
Second derivative of carbonyl region of both lipids and proteins from the control group and the other treated groups showing the underlying bands.

Absorptions due carbonyl bands were carefully investigated by differentiated spectra. Second derivative spectroscopy revealed interesting information that was masked in the original data due to variation in the absorption intensities. In Fig. 5, the two bands that discernible at 1797 and 1744 cm^-1^ are associated with _ester_C = O of the lipid moiety of retina. The higher frequency band in both AMIO and Vit. E groups were characterized by reduced band position and increased band width. The position of the second band is reduced in AMIO group while; in Vit. E treated group, it mimics the control value.

On the other hand, retinal proteins secondary structure is obviously influenced by short term administration of Amiodarone. Detection of Turns constituent and reduced contents of α-helix, β-sheet and β-turns represent the major findings as given in table 3. Vitamin E administered group pattern indicates an increased content of β-turns only while, both α-helix and β-sheet contents are mimicking the control values.

**Table 3:** Second derivative spectra of carbonyl bands of retinal lipids and proteins.

Control	Ester C = O of lipids		Amide I of protein			
			β-turns	Turns	α-helix	β-sheet
	1797 ± 2 15 ± 5 (w)	1744 ± 2 25 ± 4 (w)	1683 ± 2 10.3 ± 4 (a)		1646 ± 1 57 ± 3 (a)	1623 ± 2 28.7 ± 5 (a)
AMIO group	^†^1784 ± 3 ^†^38 ± 3 (w)	^†^1736 ± 1 30 ± 6 (w)	1680 ± 3 ^†^4.1 ± 1 (a)	1660 ± 4 38.5 ± 4 (a)	1641 ± 2 ^†^46 ± 4 (a)	1625 ± 2 ^†^11.4 ± 3 (a)
Vit. E group	^†^1780 ± 3 ^†^43 ± 3 (w)	1742 ± 1 29 ± 4 (w)	1674 ± 1 ^†^20.6 ± 3 (a)		1649 ± 2 57 ± 3 (a)	1626 ± 4 22.4 ± 3 (a)

(w) Indicate the width in cm^−1^, and (a) indicates the area percentage.

^†^Statistically significant relative to the control.

### Principal component analysis

**Fig. 6:**
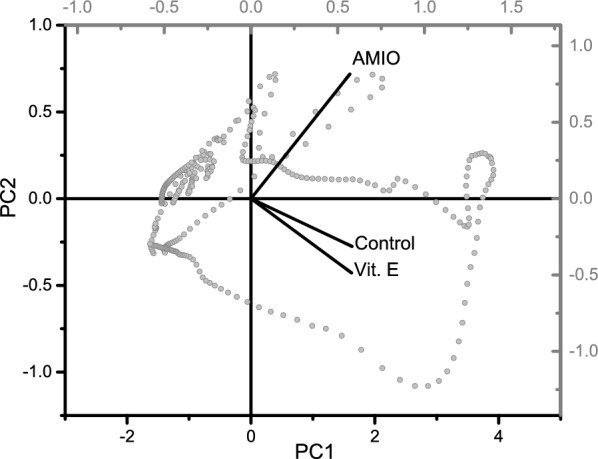
Loading plot of the principal component analysis of FTIR data for AMIO group and Vitamin E Co-administered group compared with the control group.

Principal component (PC) analysis was applied to full range of FTIR data for all groups, and the results are displayed in Fig. 6. The first two PCs cover 100% of the data where; the percentage of variance of PC1 is 94.89% and that of PC2 is 5.11%. This loading plot reveals the relationships between the studied groups. It is clear that AMIO group and Vit. E group has similar loading to PC1 but the angle between them is right angle, and both groups have different directions i.e. reverse relationship according to PC2. Comparing the two vectors of the control and Vit. E groups; it is noteworthy that they have similar loading to PC1 and the angle between them is acute angle.

## Discussion

FTIR investigation is based on the absorption of infrared light by vibrational transitions in the covalent bonds of the analyzed sample. Therefore, this analysis gives detailed information about the structure and conformation of the examined specimen^19^. Numerous studies have evidenced the accuracy of this approach in diagnostic diseases such as diabetes, cancer, and Alzheimer's disease^20^.

Full clinical effects of oral Amiodarone can be achieved after 6 weeks; and upon discontinuation of AMIO therapy, the pharmacological effects could continue for 1 to 3 months^2^. In addition, histopathological studies reveal that during AMIO therapy, intracytoplasmic deposits of Amiodarone can be found in the cornea, lens, optic nerve, and retina^21^. Other studies have shown that the prevalence of Amiodarone usage is increasing globally, especially among the elderly population^22^, and ocular changes induced by Amiodarone have also been reported. The incidence of visual disturbances in patients taking Amiodarone ranges from 1.4% to 40%. Corneal epithelial deposits are very common, occurring in 70% to 100% of patients receiving Amiodarone^11,23^.

The retinal blood vessel network is unique in that it is the only blood vessel network in the body visible through non-invasive imaging methods. Retinal fundus color imaging is a common technique used for evaluation of this vascular structure. Analyzing the structure of the retinal vessel network serves as a reliable tool for the early detection of retinopathies^24^. In this short term study, ophthalmic examinations by Fundus camera reveal no detectable clinical changes in the retinae while; the FTIR spectra demonstrated various retinal structure changes due to AMIO treatment that were noted through the absorption frequencies and bandwidth changes of many retinal functional groups. The changes observed in the NH-OH region (_str_O-H, O-H_asym,_ and O-H_sym_ vibrational bands) are related to hydrogen bonding. AMIO treatments affect retinal constituents with OH groups, and alter the molecular structure through induction of hydrogen bonding. Vitamin E was found to reduce these effects where the FTIR-pattern was different than that of AMIO group and have similarities to control pattern. In addition, Vit. E increases the order of retinal membranes through affecting the symmetric vibrational mode of both NH and OH groups. Detection of NH_sym_ band in Vit. E group is related to molecular alteration in retinal proteins^25^.

Protein misfolding is a detrimental effect that can lead to several structural/functional consequences that involving the inactivation of enzymes and protein aggregation. Typically, ocular-protein aggregation begins with forming insoluble protein fragments. Protein insolubility is dependent on the contents of β-sheet structure and α-helix as well. The association between the decreased α-helix content and increased Turns content noticed in AMIO group is indicative of a protein structure with different compositional characteristics. Taking this into consideration with the decreased content of β-turns and β-sheets, gives the impetus that the helical structure of retinal proteins becomes more folded while; the polypeptide chains were lost the ability to abruptly changing the direction due to decreased β-turns^26^. This situation is totally differed when Vit. E administered; Vit. E can buffer the undesired changes due to AMIO treatment as eluded by resuming the content of α-helix and β-sheets to their control value which is directly related to enhancing protein solubility. Not only that, but also the ability of the polypeptide chains to abruptly changing their directions is greatly enhanced as well. Moreover, the difference in Amide I/II ratio is attributed to difference in secondary structure and indicates protein rearrangement^27^. The large variation in the value of this ratio between AMIO and Vit. E groups indicate that this rearrangement is different.

Oral administration of AMIO for two weeks induce changes in the molecular structure of retinal lipids. It increases the disorder of retinal membranes; the detection of _asym_CH_3_ vibrational mode- which is mainly due to lipids with little contribution from proteins, carbohydrates and nucleic acids- at higher frequency^20,28^ and the increased concentration of double bonds of lipids within the retinal tissue; olefinic = CH band, can be used as index of unsaturation level^20,28,29^. In the same context, the increased band position and the increased vibrational motion (band narrowing) of _asym_CH_2_ also support the increased disorder as well as the formation of hydrogen bond. Administration of Vit. E for two weeks after two weeks of AMIO treatment provides protection to these alterations.

Note that, the increase in the band area ratio of _asym_CH_2_/_sym_CH_2_ in both AMIO and Vit. E reflect an increase in the lipid content^30,31^ therefore, Vit. E has no effect in this regard. This increased ratio also indicates that lipid chain length and branching is different than the control^32^ and, again no ameliorative effect of Vit. E.

There are certain requirements for biological membranes to be functioning. These requirements include barrier properties, physical characteristics and certain mobility for proteins. All these requirements are achieved by the lipid disorder which can be characterized by the vibrational frequency of _sym_CH_2_ band where; lipid disorder can be induced by structural disorder due to protein-lipid interactions or by dynamic disorder that associated with bulk lipid interactions^33^. Our results clearly indicate that retinal lipid disorder can be correlated to structural disorder as the frequency of _sym_CH_2_ band is the same in all groups.

In another context, the absorption characteristic of PO_2_ bands is a measure of the cell activities. Phosphorylation processes of retinal tissue i.e., cellular activities, is increased due to AMIO treatment and, administration of Vit. E did not alter this increase. This conclusion was derived from the intensity ratio of PO_2_ sym/asym^34^. The band narrowing observed for _asym_PO_2_ in AMIO group indicate increased motional freedom around the phosphate group in other words; increased disorder and, Vit. E has no effect in this regard.

The acyl chain packing of lipid bilayer is monitored by the bending CH_2_ vibrational band. The upshift in its frequency after AMIO treatment concomitant with reduced band width indicate changes in the lateral packing of phospholipid hydrocarbon chains and, these changes could not be contained by post administration of vitamin E.

Finally, with all these changes and discrepancies in the results, principal component analysis comes to clarify the reality about these discrepancies, that is the structural and conformational changes of the retinal tissue as a result of AMIO treatment are completely different from their control counterparts, and that vitamin E returned these changes to their control state, as the acute angle between the vectors of the control and Vit. E groups indicate a positive correlation, while the right angle between AMIO and Vit. E vectors indicate no correlation^35^.

Vitamin E supplementation provides several benefits for eye health, particularly in preventing the progression of ocular diseases such as age-related macular degeneration (AMD) and diabetic retinopathy. Studies have shown that vitamin E, especially α-tocopherol, acts as a powerful antioxidant, protecting the retina from oxidative damage and reducing the risk of developing AMD. Research indicates that antioxidant vitamins like vitamin E can slow the progression of AMD, with those at higher risk benefiting the most from these supplements^36,37^. Additionally, tocotrienol-rich vitamin E has been found to play a crucial role in preventing the progression of retinal microhaemorrhages and diabetic macular edema in patients with diabetic retinopathy, highlighting its importance in the early treatment and prevention of this condition^38^. Additionally, vitamin E levels in the retina and retinal pigment epithelium increase with age, with older individuals showing higher concentrations of vitamin E compared to younger age groups^39^. The exact optimal dose may vary based on individual health needs and age-related requirements.

In conclusion, retinal changes associated with short-term administration of AMIO were mainly due to the formation of hydrogen bonding and deterioration of retinal proteins as well. These observations were conquered and contained by vitamin E administration, and this is supported by the results of principal component analysis. Moreover, these biophysical changes at the vibrational level of the retinal tissue could not be clinically detected by Fundus examination therefore; ophthalmic examination of patients should be a prerequisite for continuation of AMIO treatment, and vitamin E supplementation is recommended. The calculated human equivalent dose^40^ relative to the animal applied dose is 32.4 mg/kg.

## Materials and methods

### Materials

Drug Cordarone®, 200 mg with active ingredient AMIO hydrochloride, was purchased from Global Napi Pharmaceuticals Company, Cairo, Egypt. Vitamin E (alpha-tocopherol acetate, 400 mg) was purchased from Pharco Pharmaceuticals Company, Cairo, Egypt. Potassium bromide powder (KBr-IR grade) was purchased from Sigma Aldrich (St. Louis, MO, USA).

### Animals and experimental design

Three groups of healthy colored rabbits (Chinchilla, 2–2.5 kg) of both sexes (male/female, 5/5) were used in this study, where each group comprises ten rabbits (20 eyes). Animals were obtained from the animal house facility at Research Institute Ophthalmology, Giza, Egypt, and were kept separately in stainless steel cages under good ventilation and 12 h light/dark cycle during the experimental period. They have free access to an adequate standard diet and water ad-libitum, and the ambient temperature was set at 25 ± 2 °C. Animals were treated according to the guidelines of using animals in ophthalmic research established by the Association of Research in Vision and Ophthalmology (ARVO), and the protocol was approved by the Research Institute of Ophthalmology ethical committee. The study is reported in accordance with ARRIVE guidelines.

The experimental design was performed as follows; rabbits were treated orally for two weeks with Amiodarone through polypropylene orogastric tube attached to 20 ml syringe. The dose was 160 mg Kg^−1^ (AMIO group). The second group was on oral administration as the AMIO group for two weeks then, received oral dose of vitamin E (100 mg Kg^−1^) for another two weeks (Vit. E group). The last group was served as the control and received 5 ml of water by gavage tube. Rabbits were sedated as a prerequisite for administration by intramuscular injection of a mixture of ketamine (80 mg/kg) and xylazine (20 mg/kg)^41^.

### Ophthalmic examination

High resolution Fundus images were taken using Topcon TRC-50EX (Japan) which is a mydriatic retinal camera incorporating digital ready features to provide complete retinal imaging including color, red-free and fluorescein angiography, with SONY DXC-950P (Japan) which is a 3CCD color video camera. The angle of coverage is 50°, 35°, and 20°.

### Sample preparation and FTIR measurement

At the end of the administration period and after the ophthalmic examination, rabbits were killed by intravenous administration of sodium pentabarbitone (30 mg Kg^−1^)^42^, and their eyes were enucleated then opened by the corneal section through the ora Serrata where the anterior segment constituents can be removed so that the retina is exposed, and can easily be obtained. Each retina was kept in a sterilized dark glass vial, flushed with dry nitrogen gas, and immediately processed for FTIR investigation (Supplementary information).

For recording the mid-infrared absorption spectrum (4000–900 cm^−1^), 10 mg of retinal tissue were mixed with 90 mg KBr powder and pressed to form a transparent KBr disk using the pressing kit provided by the manufacture. Measurements were done using an infrared spectrophotometer model Nicolet-iS5 (Thermo Fisher Scientific Inc, USA) with an effective resolution of 2 cm^-1^. The spectra were recorded under a continuous nitrogen gas flow to prevent the effect of environmental vapor (CO_2_ and H_2_O) where; one hundred and fifty interferograms were co-added. These spectra were then baseline corrected and smoothed with Savitsky–Golay function (9 points). The individual spectrum that was recorded from each studied group was averaged to obtain the group spectrum using OriginPro 2015 (64-bit) software package (Origin Lab Corporation, Northampton, MA 01,060, USA). This group spectrum is displayed in the study. Bands were carefully examined by spectral resolution techniques that comprise derivative spectroscopy or band fitting.

### Statistical analysis

Results were expressed as the mean ± standard deviation (SD). The comparison between groups was performed using one-way ANOVA (OriginPro, 2015) where the significance level was set at p < 0.05. Multivariate analysis was applied to FTIR raw data for further discrimination between the studied groups. Principal component analysis was performed using the statistical tools provided by OriginPro (2015) software.

## Supplementary Information

Supplementary Information.

## Data Availability

All data generated or analyzed during this study are included in this published article and/or its supplementary information files.
